# Frequency of HLA Alleles in a Cohort of 100 Romanian Late-Life Adults: An Academic Insight into Genetic Longevity

**DOI:** 10.3390/cimb47121018

**Published:** 2025-12-05

**Authors:** Radu-Alexandru Truică, Adriana Tălăngescu, Ion Mărunțelu, Alexandra-Elena Constantinescu, Ileana Constantinescu

**Affiliations:** 1 Faculty of Medicine, “Carol Davila” Medical University Bucharest, 020021 Bucharest, Romania; 2 Centre of Immunogenetics and Virology, Fundeni Clinical Institute, 022328 Bucharest, Romania; 3 “Emil Palade” Center of Excellence for Young People in Scientific Research (EP-CEYR), Academy of Romanian Scientists, 030167 Bucharest, Romania; 4 Academy of Romanian Scientists, 030167 Bucharest, Romania

**Keywords:** HLA, longevity, immune response, genetics, immunogenetics

## Abstract

The human leukocyte antigen (HLA) system plays a crucial role in regulating the immune response and is significant in organ transplantation, disease association studies, and population genetics. But does it influence longevity? The present study aims to explore the frequency of HLA alleles in a cohort of 100 individuals in the 65–90 age bracket from Romania, providing insights into genetic diversity and potential implications in longevity. High-resolution HLA typing was performed using next-generation sequencing (NGS) technology, allowing for precise identification of HLA alleles with a high degree of accuracy. The results reveal significant genetic diversity within the cohort, with prevalent alleles such as HLA-A*02:01:01:01, HLA-B*08:01:01:01, and DRB1*01:01:01:01 potentially influencing disease susceptibility and longevity. The study reveals the genetic diversity of HLA alleles in elderly Romanians, highlighting prevalent alleles that could be linked to longevity and disease resistance. Different results from previous research are attributed to the high-resolution analysis and small cohort size. Further studies with larger samples are needed to confirm findings and uncover their implications for healthy aging and healthcare.

## 1. Introduction

The Earth’s population is aging rapidly, with a significant increase in the number of people over 65. The WHO estimates that in less than 25 years, 20% of the planet’s population will be over 60, and by 2050, around 5% of the population will be over 80, putting pressure on public health and pension systems [1]. The UN also estimates that by 2050, one in six people will be over age 65 [2], and by 2080, people aged 65 and over will outnumber children under 18, exceeding 20% of the population [3].

The aging process is not yet fully understood, but we do know that it is a very complex process, influenced by many genetic, immunological, environmental, and lifestyle factors.

In 2000, Gudmundsson H et al. [4] published a comprehensive genealogical study involving 1531 Icelandic individuals who lived beyond the age of 94 years in men (761 cases) and 96 years in women (770 cases). The authors reconstructed detailed family trees, initially extending up to six generations (circa 1730), and in some cases tracing ancestry as far back as the year 1500. Their findings revealed a strong, sex-specific correlation of longevity among first-degree relatives, positioning them among the first to provide evidence of such a hereditary pattern of longevity in humans.

In 2002, Perls TT et al. [5] demonstrated that the siblings of centenarians have a significantly increased likelihood of achieving exceptional longevity compared to the general population. However, their study did not include an immunogenetic component.

Recent studies suggest that human longevity may be correlated with optimal functioning of the immune system [6]. An inadequate or delayed immune response allows frequent and prolonged attacks by pathogens. On the other hand, an overly strong and rapid immune response can trigger an exaggerated acute inflammatory process in response to mild aggressions, leading to prolonged periods of more or less localized inflammation. Neither of these scenarios is good to a long and healthy life.

Therefore, specific genetic determinants regulating the immune response, such as human leukocyte antigen (HLA) and killer immunoglobulin-like receptor (KIR) genes, are thought to play a key role in longevity [7,8,9].

The human leukocyte antigen (HLA) system is a crucial part of the immune system, responsible for the regulation of the immune response. The HLA system is a set of molecules on the cell surface that are essential for the immune system, and these molecules are extremely polymorphic. Due to the variability of the amino acid sequence, different HLA molecules bind differently to peptides. Therefore, certain HLA molecules are more efficient at binding and presenting certain peptides. This diversity allows the immune system to respond to a huge range of pathogens. It also plays a significant role in organ transplantation, disease association studies, and population genetics. The great diversity of HLA alleles in a population ensures that no single pathogen can decimate the entire population.

This article aims to explore the frequency of HLA alleles in a cohort of 100 elderly individuals from Romania, providing insights into the genetic diversity and potential implications for medical research and healthcare.

Romania, situated in southeastern Europe, was historically known as Dacia over 2000 years ago. The Roman Empire conquered this territory in 106 A.D. Over the centuries, due to geopolitical influences, the population has experienced ethnic influences from Hungarians, Ukrainians, Germans, Turks, Greeks, Armenians, Poles, and Russians.

Thus, the Romanian population is expected to exhibit a distinct and unique variety and frequency of HLA alleles.

First of all, we would like to mention that, so far, although there are numerous studies on the frequency of HLA alleles in the Eastern European population, there are only few studies realized by six- or eight-digit high-resolution analysis.

Ivanova R. et al. (1998) [10] identified an increased frequency of HLA-DRB1*07 alleles in a cohort of 325 centenarians. Naumova E et al. (2007 [11], 2011 [12], 2013 [13]) demonstrated the association of HLA-DRB1*11 and HLA-DRB1*16 haplotypes with longevity.

Ivanova M. [14] conducted the first high-resolution HLA allele frequency study in Eastern Europe, on a total of 102 elderly (aged 65–95 years) from Bulgaria (59 individuals), Romania (29 individuals), and Kuwait (14 individuals). Their analysis showed statistically significant increased frequencies in elderly Bulgarians of the following HLA class I alleles: HLA-A*01:01:01:01, HLA-C*07:01:02, HLA-B*35:02:01, and HLA-B*15:17:01:01. Also, HLA-A*01:01:01:01 was among the most frequent HLA alleles in elderly Romanians. The study also showed a correlation between HLA alle frequency and longevity by sex. Stratification according to gender and age in the sample of Bulgarian population tested showed that HLA-B*35:02:01 appeared as a specific marker for longevity in females, since both alleles were increased only in elderly females compared to young female controls. On the other hand, HLA-B*15:17:01:01 and HLA-C*07:01:02 were associated with longevity in men.

Comparison of HLA class II allele frequencies in elderly and young controls from Bulgarian population showed that HLA-DRB1*07:01:01, HLA-DQB1*04:02:01 and HLA-DQB1*02:57 alleles were significantly increased in elderly. Alleles HLA-DRB1*04:10:01, HLA-DQB1*05:01:04, and HLA-DQA1*03:03:01:02 were observed only in elderly individuals, and the differences were statistically significant. HLA-DRB1*07:01:01 was among the most frequent HLA alleles in elderly Romanians.

A large study conducted by Dratwa-Kuzmin M et al. [15] on a cohort of 957 individuals aged 65 to 99 years from Bulgaria, Turkey, Romania, and Poland was published in 2024. The authors noticed a correlation between HLA alleles, telomere length, and longevity. This study concluded that HLA gene frequencies show a high degree of variability between populations and a striking geographical correlation. The study concluded a correlation between the HLA-DRB1*11 and HLA-DRB1*16 allele and telomere length in the Bulgarian elderly population. For the Romanian elderly population, the HLA-C*04 and HLA-DQA1*01 alleles were associated with longer telomeres. In the Turkish elderly cohort, the HLA-A*02, HLA-B*18 and HLA-DRB1*13 alleles were associated with telomere length. In the Polish elderly cohort, the HLA-B*15 and HLA-DRB1*13 alleles were corelated with telomere length.

In 2005, Yogita G et al. [16] published a large review of HLA alleles that offer protection or susceptibility to various diseases. The authors mentioned a correlation between ankylosing spondylitis (AS) and HLA-B*27. They demonstrated that HLA-DQA1*05:01 and HLA-DQB1*02:01 are susceptibility alleles for celiac disease (CI).

They also concluded that HLA-DQA1*03:01, HLA-DQA1*05:01, HLA-DQA1*06:01, and HLA-DQB1*03:01 are protective alleles for tuberculosis (TB), while HLA-DRB1*02, HLA-DRB1*15:01, HLA-DRB1*07, HLA-DQB1*05:02, and HLA-DQA1*01:01 are susceptible alleles for TB. On the other hand, they showed that HLA-DQA1*01:02, HLA-DPB1*01:01, HLA-B*58:01, HLA-B27, HLA-B51, and HLA-B57 are protective alleles for HIV disease progression to AIDS, while the haplotype DRB1*03:01-DQA1*05:01-DQB1*02:01 is associated with disease progression.

In the case of type 1 diabetes (T1D), DQB1*03:02, DQB1*02:01, DQA1*05:01, DQA1*03:01, and haplotype DRB1*03:01-DQA1*05:01-DQB1*02:01 were the most common disease susceptibility HLA molecules, while DQB1*06:02 and DQB1*03:01 were the most common disease-protective alleles in various populations.

Regarding ankylosing spondylitis (AS), a highly heritable and common rheumatic condition, the authors concluded that HLA-B*27:05, HLA-B*27:03, and HLA-B*27:06 are protective alleles, while most of the HLA-B*27 and HLA-B*60 alleles are susceptible alleles.

The authors mention HLA-DRB1*08, DRB1*07:01, DRB1*04:03, DRB1*04:02, DRB1*01:03, DRB1*03:01, DRB1*11:02, DRB1*11:03, DRB1*13:01, DRB1*13:02, DRB1*13:04, and DRB1*04:07 and the DRB1*04:03-DQB1*0301 haplotype as protective alleles for rheumatoid arthritis (RA) and DRB1*04:05, DRB1*10:01, DRB1*09:01, the DRB1*04:01-DQB1*03:02 haplotype, DRB1*0101, DRB1*0102, DRB1*04:04, and DRB1*04:08 as susceptible alleles.

## 2. Materials and Methods

The study involved 100 elderly neurovascular patients, aged between 65 and 90 years old, from various regions of Romania. Blood samples were collected ethically with informed consent. Exclusion criteria included HCV, HBV, HIV, and neoplasms.

We thus excluded patients with a history of neoplasia or chronic HIV/HBV/HCV infections. A cohort of healthy elderly people is difficult to define and recruit. We therefore chose neurovascular patients because they represent a common and well-defined phenotype of aging. Furthermore, neurovascular history is not influenced by the effectiveness of the immune response to pathogens.

The study was conducted from 2022 to 2024.

We conducted a comparison of the HLA allele frequency between a cohort of 100 elderly individuals (51 female patients and 49 male patients) and a reference group of 100 young people (45 females and 55 males), under 35 years old, from the National Registry of Donors.

The median age for the elderly group is 69 years, with the same median for females and 68 years for males.

The median age for the reference group is 31 years, with the same median for females and 30 years for males.

High-resolution HLA typing was performed using next-generation sequencing (NGS) technology. This method allows precise identification of HLA alleles with a high degree of accuracy. The loci examined included HLA-A, HLA-B, HLA-C, HLA-DRB1, HLA-DPB1, HLA-DQA1, and HLA-DQB1.

HLA allele polymorphisms identification was performed at Fundeni Clinical Institute—Centre for Immunogenetics and Virology (Bucharest, Romania) by next-generation sequencing, using the Illumina platform (MiaFora, Immucor Germany, Bucharest, Romania).

Written consent for participation in the study and the publication of the resulting data was obtained from all participants or their relatives.

## 3. Results

### 3.1. HLA-A Locus

In the elderly cohort, the HLA-A locus showed a variety of alleles, with A*02:01:01:01 being the most common at 22.5%. Other frequent alleles were A*01:01:01:01 (15%), A*24:02:01:01 (10%), A*03:01:01:01 (8.5%), and A*11:01:01:01 (8%) [Table A1].

**Figure 1:**
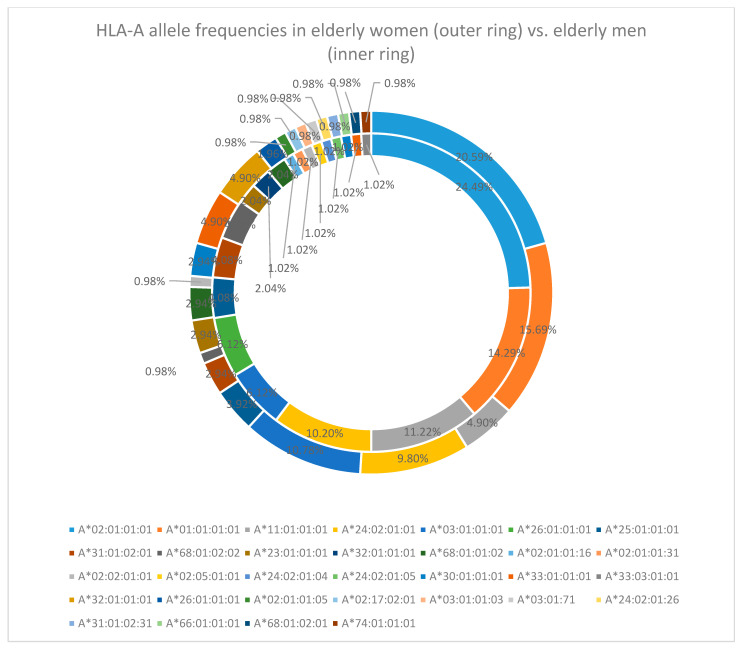
HLA-A allele frequency in the elderly cohort (women—outer ring vs. men—inner ring).

In both men and women, there is a consistency in frequencies for the first two of these alleles (HLA-A*02:01:01:01 [20.59% in women and 24.49% in men] and HLA-A*01:01:01:01 [15.69% in women and 14.29% in men]), followed by A*03:01:01:01 (10.78%) and A*24:02:01:01 (9.80%) in elderly women and by A*11:01:01:01 (11.22%) and A*24:02:01:01 (10.20%) in elderly men [Figure 1].

We also analyzed the HLA-A allele frequencies only for the 75–90 segment (36 late-life adults), and the results are similar, with A*02:01:01:01 being the most common (22.22%), followed by A*01:01:01:01 (13.89%) and A*24:02:01:01 (11.11%).

In the reference cohort of young individuals, the HLA-A locus exhibited similar results. The allele A*02:01:01:01 was the most prevalent at 32.5%, followed by A*24:02:01:01 at 11.5%, A*01:01:01:01 at 10.5%, and both A*03:01:01:01 and A*11:01:01:01 at a frequency of 8% [Table A1].

### 3.2. HLA-B Locus

At the HLA-B locus, the most common allele was B*08:01:01:01 (10%), followed by B*35:01:01:05 (8.5%), B*07:02:01:01 (5%), and B*44:02:01:01 (4.5%) [Table A1].

**Figure 2:**
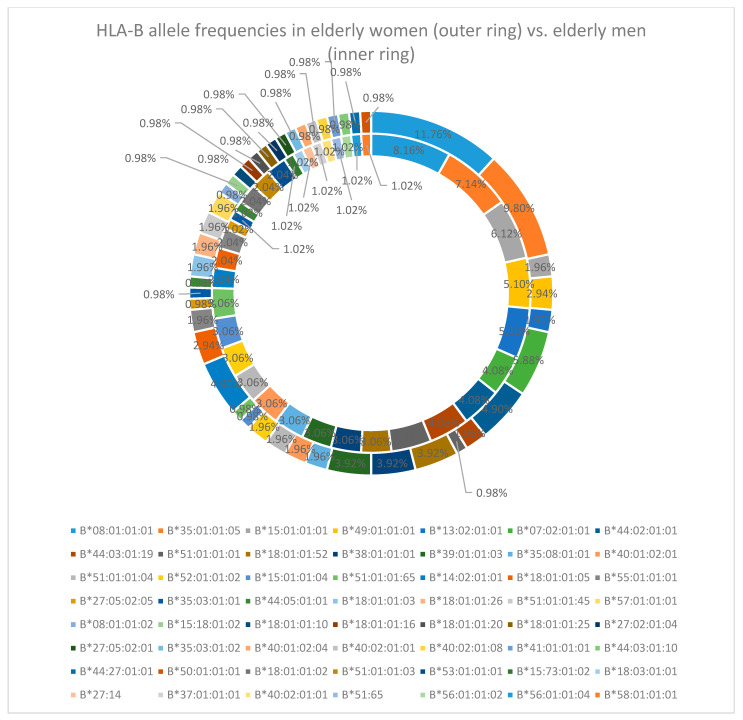
HLA-B allele frequency in the elderly cohort (women—outer ring vs. men—inner ring).

The situation for the sex-specific alleles is similar to the one for the HLA-A locus, meaning that B*08:01:01:01 (11.76% in women and 8.16% in men) and B*35:01:01:05 (9.80% in women and 7.14% in men) are the most common for both sexes, but are followed by B*07:02:01:01 (5.88%) and B*44:02:01:01 (4.90%) alleles in late-life women, while the following most frequent alleles in men are B*15:01:01:01 (6.12%), B*49:01:01:01, and B*13:02:01:01 (both with 5.10%) [Figure 2].

For the 75–90-year-old segment, the results are slightly different, with B*35:01:01:05 being the most frequent allele (11.11%), followed by B*08:01:01:01 (8.33%), while B*07:02:01:01 has a frequency of 5.56% and B*44:02:01:01 only 2.78%.

In the reference cohort, the results were different, with B*18:01:01:52 (6.5%) being the most common allele, followed by B*07:02:01:01 (6%), B*14:02:01:01 (5.5%), and both B*08:01:01:01 and B*35:01:01:05 at 5% [Table A1].

### 3.3. HLA-C Locus

For the HLA-C locus, C*12:03:01:01 was the most common allele, identified in 13.5% of the cohort. Other notable alleles included C*07:01:01:16 (11%), C*07:01:01:01 (8.5%), and C*04:01:01:01 (7.5%) [Table A1].

**Figure 3:**
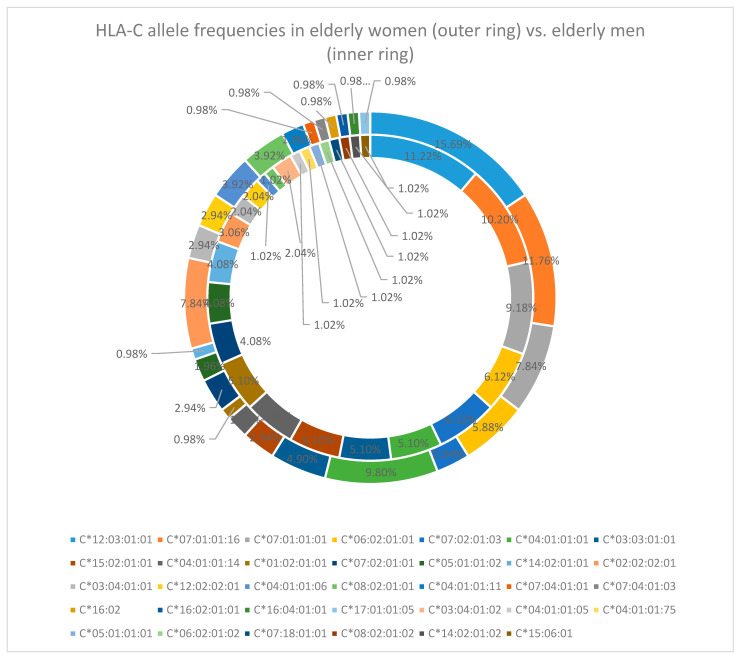
HLA-C allele frequency in the elderly cohort (women—outer ring vs. men—inner ring).

Similarly to HLA-A and HLA-B loci, if we calculate the sex-specific frequencies for the elderly cohort, we can find that C*12:03:01:01 is the most common for each sex (15.69% in females and 11.22% in males), followed by C*07:01:01:16 (11.76% and 10.20%, respectively). After this we have C*04:01:01:01 (9.80%), C*07:01:01:01, and C*02:02:02:01 (both in 7.84%) for women and C*07:01:01:01 (9.18%), C*06:02:01:01, and C*07:01:01:03 (both in 6.12%) for men [Figure 3].

For the 75–90-year-old segment the results are similar, with C*12:03:01:01 being the most frequent allele (19.44%), followed by C*07:01:01:01 (8.33%), while C*07:01:01:16 appears at only 5.56% and C*04:01:01:01 at only 2.78%.

In the reference cohort, the results were the following: C*07:01:01:16 (12.5%), C*12:03:01:01 (9.5%), C*07:02:01:03 (5.5%), C*08:02:01:01 (5.5%), and C*15:02:01:01 (5.5%) [Table A1].

### 3.4. HLA-DRB1 Locus

The HLA-DRB1 locus showed significant variability, with DRB1*01:01:01:01 being the most frequent allele (11%). DRB1*16:01:01 (10%), DRB1*03:01:01:01 (9%), and DRB1*11:04:01:01 (8.5%) were also prevalent [Table A2].

**Figure 4:**
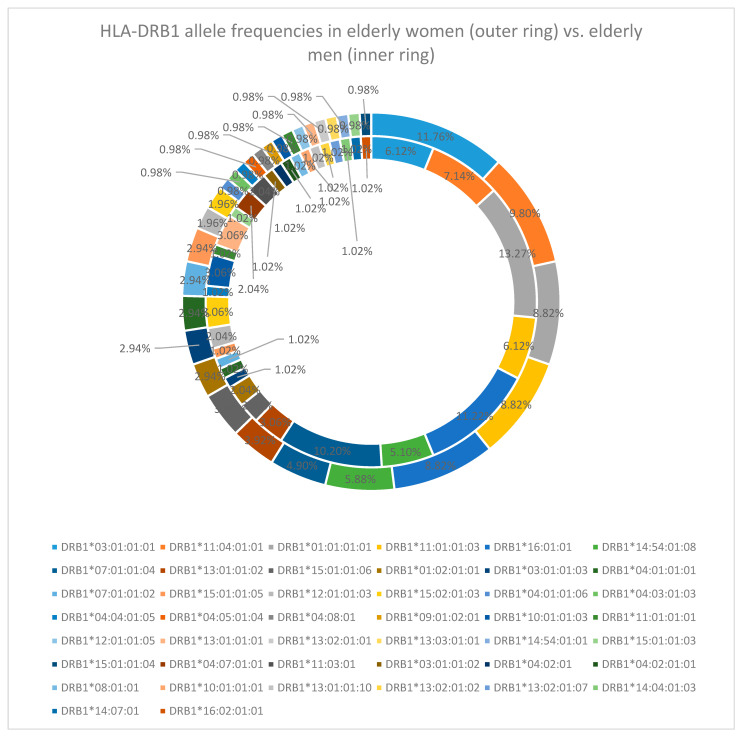
HLA-DRB1 allele frequency in the elderly cohort (women—outer ring vs. men—inner ring).

DRB1 locus has different results when performing a sex-specific analysis. In elderly women, the most frequent alleles are DRB1*03:01:01:01 (11.76%), DRB1*11:04:01:01 (9.80%), and DRB1*01:01:01:01, DRB1*11:01:01:03, and DRB1*16:01:01 (each in 8.82%). In late-life men, the most common alleles are DRB1*01:01:01:01 (13.27%), DRB1*16:01:01 (11.22%), DRB1*07:01:01:04 (10.20%), and DRB1*11:04:01:01 (7.14%), with DRB1*03:01:01:01 being found only in 6.12% of the alleles [Figure 4].

For the HLA-DRB1 locus, the reference cohort showed the following results: DRB1*11:04:01:01 (15%), DRB1*16:01:01 (8.5%), DRB1*01:01:01:01 (8%), DRB1*03:01:01:01 (7.5%), and DRB1*07:01:01:04 (7.5%) [Table A2].

### 3.5. HLA-DQB1 Locus

**Figure 5:**
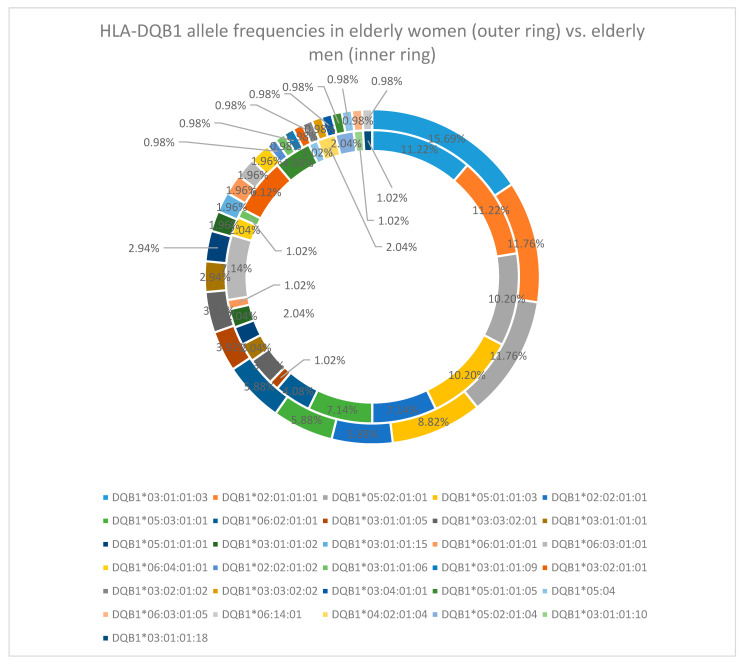
HLA-DQB1 allele frequency in the elderly cohort (women—outer ring vs. men—inner ring).

At the HLA-DQB1 locus, DQB1*03:01:01:03 was the leading allele (13.5%). Other common alleles included DQB1*02:01:01:01 (11.5%), DQB1*05:02:01:01(11%), and DQB1*05:01:01:03 (9.5%) [Table A2]. For this locus, sex-specific analysis showed no difference for the first four most common alleles. Allele DQB1*03:01:01:03 was found in 15.69% of late-life women and 11.22% of late-life men, allele DQB1*02:01:01:01 in 11.76% of women and 11.22% of men, DQB1*05:02:01:01 in 11.76% of women and 10.20% in men, and DQB1*05:01:01:03 in 8.82% of elderly women and 10.20% of elderly men [Figure 5].

In the reference cohort of young individuals, the HLA-DQB1 locus exhibited the following results: the allele DQB1*03:01:01:03 was the most prevalent at 13.5%, followed by DQB1*05:02:01:02 (10.5%), DQB1*03:02:01:01 (9%), and DQB1*03:01:01:09 (8.5%) [Table A2].

### 3.6. HLA-DQA1 Locus

At the HLA-DQA1 locus, the predominant allele was DQA1*05:05:01:01, with a frequency of 11.5%. Other frequently observed alleles included DQA1*01:02:02:01 (9.5%), DQA1*05:01:01:02 (9.5%), and DQA1*02:01:01:01 (9%) [Table A2].

**Figure 6:**
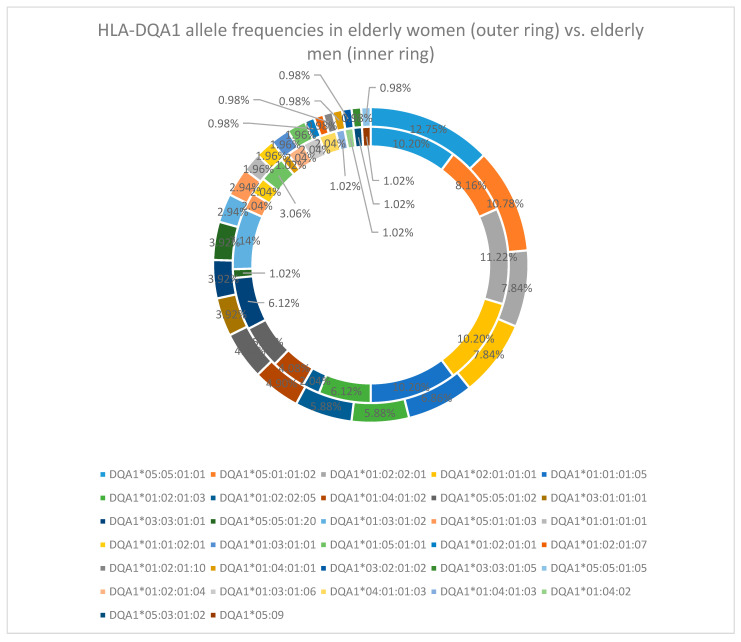
HLA-DQA1 allele frequency in the elderly cohort (women—outer ring vs. men—inner ring).

The sex-specific analysis revealed the following: in late-life women, the most frequent HLA-DQA1 alleles were DQA1*05:05:01:01 (12.75%), DQA1*05:01:01:02 (10.78%), DQA1*01:02:02:01, and DQA1*02:01:01:01 (both in 7.84%), while in late-life men, the most common HLA-DQA1 allele was DQA1*01:02:02:01 (11.22%), followed by DQA1*05:05:01:01, DQA1*02:01:01:01, and DQA1*01:01:01:05 (all three in 10.20%), with DQA1*05:01:01:02 in only 8.16% [Figure 6].

In the reference cohort, the result was comparable, with DQA1*05:05:01:01 (14.5%) being the most common allele, followed by DQA1*01:02:02:01 (9.5%), DQA1*05:05:01:02 (9.5%), and DQA1*02:01:01:01 (9%) [Table A2].

### 3.7. HLA-DPB1 Locus

At the HLA-DPB1 locus, the predominant allele was DPB1*04:01:01:06, with a frequency of 22.5%. Other frequently observed alleles included DPB1*02:01:02:05 (17.5%), DPB1*04:01:01:05 (11.5%), and DPB1*04:02:01:02 (7.5%) [Table A2].

**Figure 7:**
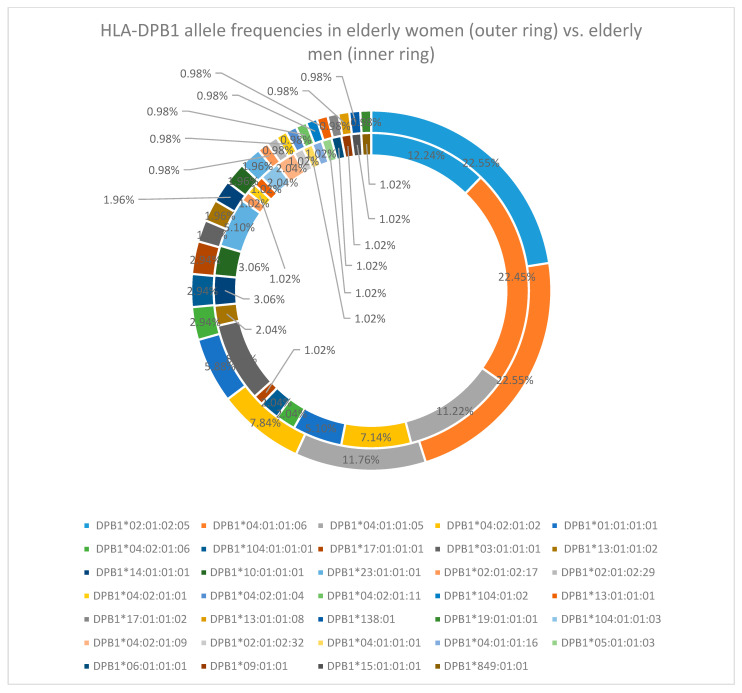
HLA-DPB1 allele frequency in the elderly cohort (women—outer ring vs. men—inner ring).

The sex-specific analysis, just like for the HLA-DQB1 locus, did not reveal any changes in the frequencies, with DPB1*04:01:01:06 being the most frequent (22.55% in women and 22.45% in men), followed by DPB1*02:01:02:05 (22.55% in women and 12.24% in men), DPB1*04:01:01:05 (11.76% in women and 11.22% in men), and DPB1*04:02:01:02 (7.84% in women and 7.14% in men) [Figure 7].

For the HLA-DPB1 locus, the results were almost the same. DPB1*04:01:01:06 was the most frequent allele (22%), followed by DPB1*02:01:02:05 (19.5%), DPB1*04:02:01:02 (11.5%), DPB1*03*01*01*01 (8%), and DPB1*04:01:01:05 (7%) [Table A2].

## 4. Discussion

The present study offers a comprehensive overview of HLA allele frequencies in a cohort of 100 Romanian individuals aged 65 years and older. The observed HLA allele frequencies in this Romanian elderly cohort demonstrates genetic diversity and presents slightly different results compared to previous studies.

For the HLA-A loci, the most frequent allele in our cohort was A*02:01:01:01 (22.5%), followed by A*01:01:01:01 (15%). Previous studies identified HLA-A*01:01:01:01 [12] and HLA-A*02 [13] as the most common HLA-A alleles.

Regarding the HLA-B loci, the predominant allele in our cohort was B*08:01:01:01 (10%), followed by B*35:01:01:05 (8.5%). In contrast, earlier studies reported HLA-B*35:02:01, HLA-B*15:17:01:01 [12], and HLA-B*18 [13] as the most frequent.

Concerning the HLA-C loci, C*12:03:01:01 was the most common allele (13.5%), followed by C*07:01:01:16 (11%) and C*07:01:01:01 (8.5%). Literature reports indicate HLA-C*07:01:02 [12] and HLA-C*04 [13] as the most frequent alleles.

For the HLA-DRB1 loci, DRB1*01:01:01:01 was the most common allele identified in our study (11%). Previous studies cited HLA-DRB1*07:01:01, DRB1*04:10:01 [12], and HLA-DRB1*13 [13] as the most prevalent.

At the HLA-DQB1 locus, DQB1*03:01:01:03 was the leading allele (13.5%) in our study, whereas the literature reports HLA-DQB1*04:02:01 and HLA-DQB1*02:57 [12] as the most common alleles.

At the HLA-DQA1 locus, the predominant allele was DQA1*05:05:01:01, with a frequency of 11.5% in our study. However, previous studies indicated HLA-DQA1*03:03:01:02 [12] as the predominant allele.

The sex-specific analysis in the late-life cohort does not reveal any remarkable changes. The only locus with atypical results is HLA-DQA1. For the rest of the loci, the first three most frequent alleles are the same while performing sex classification.

The alleles that we identified as most common are not among the few alleles that are known to be protective. The reasons could be that there are no comprehensive high-resolution HLA studies of large populations. We did find a high frequency of DRB1*03:01:01:01 (11.76% in elderly women and 6.12% in elderly men), and it is known that the DRB1*03:01 allele is considered a protective allele for rheumatoid arthritis, but also a susceptible allele for type 1 diabetes or HIV progression to AIDS; however, with these studies being low-resolution, we do not know what impact and role each allele has. We also identified the HLA-DQB1*03:01:01:03 allele in 15.69% of elderly women and in 11.22% on elderly men. DQB1*03:01 is a known protective allele for tuberculosis and type 1 diabetes. We also identified HLA-DQA1*01:02:02:01 in 7.84% of late-life women and 11.22% of late-life men. The DQA1*01:02 allele is considered to be protective for both type 1 diabetes and HIV progression to AIDS.

The prevalent alleles, such as HLA-A*02:01:01:01, HLA-B*08:01:01:01, HLA-B*35:01:01:05, HLA-C*12:03:01:01, HLA-DQB1*03:01:01:03, or HLA-DQA1*01:02:02:01, might enhance immune response or protect against age-related diseases, potentially contributing to longevity. Understanding the effects of these alleles on both disease resistance and lifespan could provide insights into factors promoting healthy aging and longevity. Notably, other HLA loci, such as HLA-DRB1*01 and HLA-DRB1*16, are frequently associated with longevity. Analyzing the presence and impact of these alleles within our cohort could further elucidate their roles in promoting longevity.

The differences in allele frequencies might reflect the unique genetic background of the Romanian population, shaped by historical migrations and foreign intervention. Romania’s geographic position at the crossroads of Central and Eastern Europe may have contributed to a distinct HLA profile.

The present study is limited by the cohort size, which may not fully represent the broader Romanian population. Future work should expand to larger cohorts and explore HLA haplotype frequencies to gain deeper insights.

Insights from this broader genetic landscape can aid in identifying specific HLA profiles that contribute to a healthier and longer life.

## 5. Conclusions

This study on the frequency of HLA alleles in a cohort of 100 elderly Romanian individuals reveals significant genetic diversity and presents slightly different results compared to previous studies. The prevalent alleles, such as HLA-A*02:01:01:01, HLA-B*08:01:01:01, and DRB1*01:01:01:01, may influence disease susceptibility and play a role in longevity. Understanding the effects of these alleles on both disease resistance and lifespan could offer insights into factors promoting healthy aging and longevity.

It is important to note that our results may differ from other studies for two main reasons. Firstly, this is the first high-resolution HLA study with eight digits, providing a more detailed and accurate analysis of HLA allele frequencies. Secondly, our cohort was relatively small, and the results could differ in assessments involving larger cohorts. Further research with larger sample sizes is needed to validate these findings and explore the potential implications for medical research and healthcare. These should be considered when interpreting the findings and their implications.

The potential impact of these findings is significant, as they could contribute to a better understanding of the genetic factors influencing longevity and disease resistance. Insights from this broader genetic landscape can aid in identifying specific HLA profiles that contribute to a healthier and longer life.

## Data Availability

The original contributions presented in this study are included in the article. Further inquiries can be directed to the corresponding author(s).

## Abbreviations

The following abbreviations are used in this manuscript:

AS	Ankylosing spondylitis
CI	Celiac disease
HLA	Human Leukocyte Antigen
KIR	Killer Immunoglobulin-like Receptor
NGS	Next-Generation Sequencing
RA	Rheumatoid arthritis
T1B	Type 1 diabetes
TB	Tuberculosis

## Appendix A

Here are all the frequencies obtained for each locus for the elderly cohort and for the young cohort.

**Table A1:** Class I HLA alleles by frequencies in the elderly cohort vs. the reference cohort.

**Class I HLA Alleles**			
**HLA-A**			
**Elderly Group**		**Reference Group**	
**HLA-A**	**Frequency**	**HLA-A**	**Frequency**
A*02:01:01:01	22.50%	A*02:01:01:01	32.50%
A*01:01:01:01	15.00%	A*24:02:01:01	11.50%
A*24:02:01:01	10.00%	A*01:01:01:01	10.50%
A*03:01:01:01	8.50%	A*03:01:01:01	6.00%
A*11:01:01:01	8.00%	A*11:01:01:01	6.00%
A*25:01:01:01	4.00%	A*26:01:01:01	5.50%
A*26:01:01:01	4.00%	A*32:01:01:01	3.50%
A*31:01:02:01	3.50%	A*25:01:01:01	3.00%
A*32:01:01:01	3.50%	A*23:01:01:01	2.50%
A*33:01:01:01	3.00%	A*33:01:01:01	2.50%
A*23:01:01:01	2.50%	A*29:02:01:01	1.50%
A*68:01:01:02	2.50%	A*33:03:01:01	1.50%
A*68:01:02:02	2.50%	A*68:01:01:02	1.50%
A*30:01:01:01	2.00%	A*02:01:01:159	1.00%
A*02:02:01:01	1.00%	A*02:02:01:01	1.00%
A*02:01:01:05	0.50%	A*24:02:01:04	1.00%
A*02:01:01:16	0.50%	A*24:02:01:05	1.00%
A*02:01:01:31	0.50%	A*26:01:01:02	1.00%
A*02:05:01:01	0.50%	A*30:01:01:01	1.00%
A*02:17:02:01	0.50%	A*31:01:02:01	1.00%
A*03:01:01:03	0.50%	A*01:01:01:03	0.50%
A*03:01:71	0.50%	A*02:01:01:16	0.50%
A*24:02:01:04	0.50%	A*02:05:01:01	0.50%
A*24:02:01:05	0.50%	A*02:11:01:01	0.50%
A*24:02:01:26	0.50%	A*03:01:71:01	0.50%
A*31:01:02:31	0.50%	A*03:02:01:01	0.50%
A*33:03:01:01	0.50%	A*30:04:01:01	0.50%
A*66:01:01:01	0.50%	A*68:01:02:01	0.50%
A*68:01:02:01	0.50%	A*68:01:02:02	0.50%
A*74:01:01:01	0.50%	A*68:02:01:01	0.50%
	100.00%		100.00%
**HLA-B**			
**Elderly Group**		**Reference Group**	
**HLA-B**	**Frequency**	**HLA-B**	**Frequency**
B*08:01:01:01	10.00%	B*18:01:01:52	6.50%
B*35:01:01:05	8.50%	B*07:02:01:01	6.00%
B*07:02:01:01	5.00%	B*14:02:01:01	5.50%
B*44:02:01:01	4.50%	B*08:01:01:01	5.00%
B*15:01:01:01	4.00%	B*35:01:01:05	5.00%
B*49:01:01:01	4.00%	B*44:02:01:01	4.50%
B*13:02:01:01	3.50%	B*51:01:01:04	4.50%
B*14:02:01:01	3.50%	B*15:01:01:01	4.00%
B*18:01:01:52	3.50%	B*18:01:01:02	4.00%
B*38:02:01:01	3.50%	B*38:01:01:01	4.00%
B*39:01:01:03	3.50%	B*35:08:01:01	2.50%
B*44:03:01:19	3.00%	B*49:01:01:01	2.50%
B*18:01:01:05	2.50%	B*55:01:01:01	2.50%
B*35:08:01:01	2.50%	B*18:01:01:26	2.00%
B*40:01:02:01	2.50%	B*35:03:01:01	2.00%
B*51:01:01:01	2.50%	B*37:01:01:01	2.00%
B*51:01:01:04	2.50%	B*44:03:01:01	2.00%
B*52:01:01:02	2.50%	B*58:01:01:01	2.00%
B*57:01:01:01	2.50%	B*13:02:01:01	1.50%
B*51:01:01:65	2.00%	B*35:03:01:03	1.50%
B*55:01:01:01	2.00%	B*39:06:02:01	1.50%
B*18:01:01:02	1.00%	B*41:01:01:01	1.50%
B*18:01:01:03	1.00%	B*51:01:01:45	1.50%
B*18:01:01:26	1.00%	B*57:01:01:01	1.50%
B*27:05:02:05	1.00%	B*18:01:01:05	1.00%
B*35:03:01:01	1.00%	B*27:02:01:01	1.00%
B*40:02:01:01	1.00%	B*27:02:01:04	1.00%
B*44:05:01:01	1.00%	B*44:05:01:01	1.00%
B*51:01:01:03	1.00%	B*44:27:01:01	1.00%
B*51:01:01:45	1.00%	B*48:01:01:01	1.00%
B*53:01:01:01	1.00%	B*51:01:01:03	1.00%
B*08:01:01:02	0.50%	B*56:01:01:03	1.00%
B*15:01:01:01	0.50%	B*07:02:01:01	0.50%
B*15:18:01:02	0.50%	B*13:02:01:08	0.50%
B*15:73:01:02	0.50%	B*14:01:01:01	0.50%
B*18:01:01:10	0.50%	B*15:08:01:01	0.50%
B*18:01:01:16	0.50%	B*18:01:01:01	0.50%
B*18:01:01:20	0.50%	B*18:01:01:16	0.50%
B*18:01:01:25	0.50%	B*18:01:01:28	0.50%
B*18:03:01:01	0.50%	B*18:01:01:33	0.50%
B*27:02:01:04	0.50%	B*35:01:01:01	0.50%
B*27:05:02:01	0.50%	B*35:01:01:18	0.50%
B*27:14	0.50%	B*35:01:01:26	0.50%
B*35:03:01:02	0.50%	B*35:02:01:01	0.50%
B*37:01:01:01	0.50%	B*35:02:01:02	0.50%
B*40:01:02:04	0.50%	B*39:01:01:03	0.50%
B*40:02:01:08	0.50%	B*39:01:01:05	0.50%
B*41:01:01:01	0.50%	B*40:01:02:01	0.50%
B*44:03:01:10	0.50%	B*40:01:02:35	0.50%
B*44:27:01:01	0.50%	B*40:02:01:01	0.50%
B*50:01:01:01	0.50%	B*40:02:01:18	0.50%
B*51:65	0.50%	B*40:06:01:04	0.50%
B*56:01:01:02	0.50%	B*40:06:01:06	0.50%
B*56:01:01:04	0.50%	B*44:03:01:19	0.50%
B*58:01:01:01	0.50%	B*50:01:01:01	0.50%
	100.00%	B*51:01:01:01	0.50%
		B*51:01:01:06	0.50%
		B*51:01:01:11	0.50%
		B*51:01:01:59	0.50%
		B*51:01:01:60	0.50%
		B*51:08:01:01	0.50%
		B*52:01:01:01	0.50%
		B*52:01:01:02	0.50%
		B*56:01:01:02	0.50%
		B*58:01:01:01	0.50%
			100.00%
**HLA-C**			
**Elderly Group**		**Reference Group**	
**HLA-C**	**Frequency**	**HLA-C**	**Frequency**
C*12:03:01:01	13.50%	C*07:01:01:16	12.50%
C*07:01:01:16	11.00%	C*12:03:01:01	9.50%
C*07:01:01:01	8.50%	C*07:02:01:03	5.50%
C*04:01:01:01	7.50%	C*08:02:01:01	5.50%
C*06:02:01:01	6.00%	C*15:02:01:01	5.50%
C*02:02:02:01	5.00%	C*01:02:01:01	5.00%
C*03:03:01:01	5.00%	C*02:02:02:01	5.00%
C*07:02:01:03	4.50%	C*03:03:01:01	4.50%
C*15:02:01:01	4.00%	C*04:01:01:06	4.50%
C*04:01:01:14	3.50%	C*07:01:01:01	4.50%
C*07:02:01:01	3.50%	C*03:04:01:01	4.00%
C*01:02:01:01	3.00%	C*06:02:01:01	3.50%
C*05:01:01:02	3.00%	C*04:01:01:01	3.00%
C*03:04:01:01	2.50%	C*04:01:01:14	2.50%
C*04:01:01:06	2.50%	C*05:01:01:02	2.50%
C*08:02:01:01	2.50%	C*07:02:01:01	1.50%
C*12:02:02:01	2.50%	C*14:02:01:01	1.50%
C*14:02:01:01	2.50%	C*16:01:01:01	1.50%
C*03:04:01:02	1.00%	C*16:04:01:01	1.50%
C*04:01:01:11	1.00%	C*02:02:02:03	1.00%
C*02:02:02:02	0.50%	C*03:02:02:03	1.00%
C*04:01:01:05	0.50%	C*06:02:01:02	1.00%
C*04:01:01:75	0.50%	C*07:04:01:01	1.00%
C*05:01:01:01	0.50%	C*07:18:01:01	1.00%
C*06:02:01:02	0.50%	C*12:02:02:01	1.00%
C*07:04:01:01	0.50%	C*16:02:01:01	1.00%
C*07:04:01:03	0.50%	C*17:01:01:05	1.00%
C*07:18:01:01	0.50%	C*02:02:02:20	0.50%
C*08:02:01:02	0.50%	C*03:02:02:04	0.50%
C*14:02:01:02	0.50%	C*04:01:01:05	0.50%
C*15:06:01	0.50%	C*04:01:01:08	0.50%
C*16:02	0.50%	C*05:01:01:01	0.50%
C*16:02:01:01	0.50%	C*05:01:01:45	0.50%
C*16:04:01:01	0.50%	C*07:01:01:04	0.50%
C*17:01:01:05	0.50%	C*07:01:01:09	0.50%
	100.00%	C*07:01:01:83	0.50%
		C*07:01:01:92	0.50%
		C*07:783	0.50%
		C*08:02:01:02	0.50%
		C*08:03:01	0.50%
		C*08:03:01:01	0.50%
		C*12:03:01:09	0.50%
		C*12:03:06	0.50%
		C*16:01:01:02	0.50%
			100.00%

**Table A2:** Class II HLA alleles by frequencies in the elderly cohort vs. the reference cohort.

**Class II HLA Alleles**			
**HLA-DRB1**			
**Elderly Group**		**Reference Group**	
**HLA-DRB1**	**Frequency**	**HLA-DRB1**	**Frequency**
DRB1*01:01:01:01	11.00%	DRB1*11:04:01:01	15.00%
DRB1*16:01:01	10.00%	DRB1*16:01:01	8.50%
DRB1*03:01:01:01	9.00%	DRB1*01:01:01:01	8.00%
DRB1*11:04:01:01	8.50%	DRB1*03:01:01:01	7.50%
DRB1*07:01:01:04	7.50%	DRB1*07:01:01:04	7.50%
DRB1*11:01:01:03	7.50%	DRB1*11:01:01:03	4.50%
DRB1*14:54:01:08	5.50%	DRB1*13:01:01:01	4.50%
DRB1*13:01:01:02	3.50%	DRB1*15:01:01:03	3.50%
DRB1*15:01:01:06	3.00%	DRB1*10:01:01:03	3.00%
DRB1*01:02:01:01	2.50%	DRB1*11:03:01	2.50%
DRB1*15:02:01:03	2.50%	DRB1*04:01:01:01	2.00%
DRB1*03:01:01:03	2.00%	DRB1*04:03:01:03	2.00%
DRB1*04:01:01:01	2.00%	DRB1*04:04:01:05	2.00%
DRB1*07:01:01:02	2.00%	DRB1*04:05:01:04	2.00%
DRB1*10:01:01:03	2.00%	DRB1*13:03:01:01	2.00%
DRB1*12:01:01:03	2.00%	DRB1*04:01:01:06	1.50%
DRB1*13:01:01:01	2.00%	DRB1*04:02:01	1.50%
DRB1*15:01:01:05	2.00%	DRB1*12:01:01:04	1.50%
DRB1*04:04:01:05	1.00%	DRB1*13:01:01:02	1.50%
DRB1*04:07:01:01	1.00%	DRB1*14:54:01:08	1.50%
DRB1*11:01:01:01	1.00%	DRB1*15:01:01:05	1.50%
DRB1*11:03:01	1.00%	DRB1*01:02:01:01	1.00%
DRB1*15:01:01:03	1.00%	DRB1*07:01:01:01	1.00%
DRB1*03:01:01:02	0.50%	DRB1*08:01:01	1.00%
DRB1*04:01:01:06	0.50%	DRB1*12:01:01:03	1.00%
DRB1*04:02:01	0.50%	DRB1*13:02:01:02	1.00%
DRB1*04:02:01:01	0.50%	DRB1*13:02:01:03	1.00%
DRB1*04:03:01:03	0.50%	DRB1*15:01:01:06	1.00%
DRB1*04:05:01:04	0.50%	DRB1*15:02:01:03	1.00%
DRB1*04:08:01	0.50%	DRB1*16:02:01:02	1.00%
DRB1*08:01:01	0.50%	DRB1*01:02:01:03	0.50%
DRB1*09:01:02:01	0.50%	DRB1*01:03:01	0.50%
DRB1*10:01:01:01	0.50%	DRB1*03:01:01:03	0.50%
DRB1*12:01:01:05	0.50%	DRB1*04:03:01:01	0.50%
DRB1*13:01:01:10	0.50%	DRB1*04:07:01:01	0.50%
DRB1*13:02:01:01	0.50%	DRB1*04:08:01	0.50%
DRB1*13:02:01:02	0.50%	DRB1*07:01:01:02	0.50%
DRB1*13:02:01:07	0.50%	DRB1*11:01:01:01	0.50%
DRB1*13:03:01:01	0.50%	DRB1*12:01:01:05	0.50%
DRB1*14:04:01:03	0.50%	DRB1*13:01:01:09	0.50%
DRB1*14:07:01	0.50%	DRB1*13:02:01:07	0.50%
DRB1*14:54:01:01	0.50%	DRB1*13:02:01:09	0.50%
DRB1*15:01:01:04	0.50%	DRB1*14:04:01:01	0.50%
DRB1*16:02:01:01	0.50%	DRB1*14:04:01:03	0.50%
	100.00%	DRB1*16:02:01:04	0.50%
			100.00%
**HLA-DQB1**			
**Elderly Group**		**Reference Group**	
**HLA-DQB1**	**Frequency**	**HLA-DQB1**	**Frequency**
DQB1*03:01:01:03	13.50%	DQB1*03:01:01:03	13.50%
DQB1*02:01:01:01	11.50%	DQB1*05:02:01:01	10.50%
DQB1*05:02:01:01	11.00%	DQB1*03:02:01:01	9.00%
DQB1*05:01:01:03	9.50%	DQB1*03:01:01:09	8.50%
DQB1*02:02:01:01	6.50%	DQB1*02:01:01:01	8.00%
DQB1*05:03:01:01	6.50%	DQB1*02:02:01:01	8.00%
DQB1*06:02:01:01	5.00%	DQB1*05:01:01:03	8.00%
DQB1*06:03:01:01	4.50%	DQB1*06:03:01:01	6.50%
DQB1*03:02:01:01	3.50%	DQB1*05:03:01:01	3.50%
DQB1*03:03:02:01	3.50%	DQB1*05:01:01:05	3.00%
DQB1*03:01:01:01	2.50%	DQB1*06:02:01:01	3.00%
DQB1*03:01:01:05	2.50%	DQB1*03:01:01:02	2.50%
DQB1*05:01:01:01	2.50%	DQB1*03:01:01:05	2.00%
DQB1*05:01:01:05	2.50%	DQB1*06:04:01:01	2.00%
DQB1*03:01:01:02	2.00%	DQB1*03:01:01:01	1.50%
DQB1*06:04:01:01	2.00%	DQB1*03:01:01:10	1.00%
DQB1*06:01:01:01	1.50%	DQB1*03:03:02:01	1.00%
DQB1*03:01:01:06	1.00%	DQB1*04:02:01:04	1.00%
DQB1*03:01:01:15	1.00%	DQB1*05:01:01:01	1.00%
DQB1*04:02:01:04	1.00%	DQB1*05:02:01:04	1.00%
DQB1*05:02:01:04	1.00%	DQB1*06:09:01:01	1.00%
DQB1*05:04	1.00%	DQB1*02:02:01:02	0.50%
DQB1*02:02:01:02	0.50%	DQB1*03:02:01:02	0.50%
DQB1*03:01:01:09	0.50%	DQB1*03:04:01:02	0.50%
DQB1*03:01:01:10	0.50%	DQB1*03:05:01	0.50%
DQB1*03:01:01:18	0.50%	DQB1*05:01:01:11	0.50%
DQB1*03:02:01:02	0.50%	DQB1*05:04	0.50%
DQB1*03:03:02:02	0.50%	DQB1*06:01:01:01	0.50%
DQB1*03:04:01:01	0.50%	DQB1*06:02:01:03	0.50%
DQB1*06:03:01:05	0.50%	DQB1*06:03:01:05	0.50%
DQB1*06:14:01	0.50%		100.00%
	100.00%		
**HLA-DQA1**			
**Elderly Group**		**Reference Group**	
**HLA-DQA1**	**Frequency**	**HLA-DQA1**	**Frequency**
DQA1*05:05:01:01	11.50%	DQA1*05:05:01:01	14.50%
DQA1*01:02:02:01	9.50%	DQA1*01:02:02:01	9.50%
DQA1*05:01:01:02	9.50%	DQA1*05:05:01:02	9.50%
DQA1*02:01:01:01	9.00%	DQA1*02:01:01:01	9.00%
DQA1*01:01:01:05	8.50%	DQA1*03:01:01:01	8.50%
DQA1*01:02:01:03	6.00%	DQA1*01:01:01:05	8.00%
DQA1*01:03:01:02	5.00%	DQA1*01:03:01:02	6.50%
DQA1*05:05:01:02	5.00%	DQA1*05:01:01:02	6.00%
DQA1*01:04:01:02	4.50%	DQA1*01:02:01:03	3.50%
DQA1*03:01:01:01	4.50%	DQA1*03:03:01:01	3.50%
DQA1*01:02:02:05	4.00%	DQA1*05:05:01:20	3.50%
DQA1*01:05:01:01	2.50%	DQA1*01:02:01:04	3.00%
DQA1*03:03:01:01	2.50%	DQA1*01:05:01:01	3.00%
DQA1*05:01:01:03	2.50%	DQA1*01:01:02:02	1.00%
DQA1*05:01:01:20	2.50%	DQA1*01:02:01:01	1.00%
DQA1*01:01:02:01	2.00%	DQA1*01:02:01:05	1.00%
DQA1*04:01:01:03	1.00%	DQA1*01:02:02:05	1.00%
DQA1*01:02:01:04	1.00%	DQA1*01:03:01:01	1.00%
DQA1*01:04:01:01	1.00%	DQA1*01:04:01:02	1.00%
DQA1*01:03:01:06	1.00%	DQA1*01:04:02	1.00%
DQA1*01:03:01:01	1.00%	DQA1*04:01:01:03	1.00%
DQA1*01:01:01:01	1.00%	DQA1*05:01:01:01	1.00%
DQA1*01:02:01:01	0.50%	DQA1*05:01:01:03	1.00%
DQA1*01:02:01:07	0.50%	DQA1*01:01:02:01	0.50%
DQA1*05:09	0.50%	DQA1*01:02:01:10	0.50%
DQA1*01:02:01:10	0.50%	DQA1*01:64	0.50%
DQA1*01:04:02	0.50%	DQA1*03:03:01:05	0.50%
DQA1*03:02:01:02	0.50%		100.00%
DQA1*03:03:01:05	0.50%		
DQA1*05:03:01:02	0.50%		
DQA1*05:05:01:05	0.50%		
DQA1*01:04:01:03	0.50%		
	100.00%		
**HLA-DPB1**			
**Elderly Group**		**Reference Group**	
**HLA-DPB1**	**Frequency**	**HLA-DPB1**	**Frequency**
DPB1*04:01:01:06	22.50%	DPB1*04:01:01:06	22.00%
DPB1*02:01:02:05	17.50%	DPB1*02:01:02:05	19.50%
DPB1*04:01:01:05	11.50%	DPB1*04:02:01:02	11.50%
DPB1*04:02:01:02	7.50%	DPB1*03:01:01:01	8.00%
DPB1*01:01:01:01	5.50%	DPB1*04:01:01:05	7.00%
DPB1*03:01:01:01	5.00%	DPB1*10:01:01:01	3.50%
DPB1*23:01:01:01	3.50%	DPB1*11:01:01:01	3.50%
DPB1*04:02:01:06	2.50%	DPB1*01:01:01:01	3.00%
DPB1*10:01:01:01	2.50%	DPB1*04:02:01:01	3.00%
DPB1*14:01:01:01	2.50%	DPB1*04:02:01:06	2.50%
DPB1*104:01:01:01	2.50%	DPB1*05:01:01:01	2.00%
DPB1*13:01:01:02	2.00%	DPB1*23:01:01:01	2.00%
DPB1*17:01:01:01	2.00%	DPB1*06:01:01:01	1.50%
DPB1*02:01:02:17	1.00%	DPB1*02:01:02:17	1.00%
DPB1*13:01:01:01	1.00%	DPB1*04:02:01:08	1.00%
DPB1*104:01:01:03	1.00%	DPB1*04:02:01:19	1.00%
DPB1*04:02:01:09	1.00%	DPB1*13:01:01:01	1.00%
DPB1*04:02:01:01	1.00%	DPB1*13:01:01:02	1.00%
DPB1*02:01:02:29	0.50%	DPB1*15:01:01:01	1.00%
DPB1*02:01:02:32	0.50%	DPB1*17:01:01:01	1.00%
DPB1*04:01:01:01	0.50%	DPB1*104:01:01:01	1.00%
DPB1*04:01:01:16	0.50%	DPB1*02:01:02:44	0.50%
DPB1*849:01:01	0.50%	DPB1*03:01:01:09	0.50%
DPB1*04:02:01:04	0.50%	DPB1*04:01:01:03	0.50%
DPB1*04:02:01:11	0.50%	DPB1*04:01:01:41	0.50%
DPB1*138:01	0.50%	DPB1*14:01:01:01	0.50%
DPB1*06:01:01:01	0.50%	DPB1*59:01	0.50%
DPB1*09:01:01	0.50%		100.00%
DPB1*13:01:01:08	0.50%		
DPB1*15:01:01:01	0.50%		
DPB1*17:01:01:02	0.50%		
DPB1*19:01:01:01	0.50%		
DPB1*104:01:02	0.50%		
DPB1*05:01:01:03	0.50%		
	100.00%		
